# Novelty seeking is related to individual risk preference and brain activation associated with risk prediction during decision making

**DOI:** 10.1038/srep10534

**Published:** 2015-06-11

**Authors:** Ying Wang, Ying Liu, Lizhuang Yang, Feng Gu, Xiaoming Li, Rujing Zha, Zhengde Wei, Yakun Pei, Peng Zhang, Yifeng Zhou, Xiaochu Zhang

**Affiliations:** 1 Chinese Academy of Sciences Key Laboratory of Brain Function and Disease, and School of Life Sciences, University of Science and Technology of China, Hefei, Anhui Province, 230026, China; 2 Provincial Hospital Affiliated to Anhui Medical University, Hefei, Anhui 230001, China; 3 Department of Medical Psychology, Anhui Medical University, Hefei, Anhui 230032, China; 4 State Key Laboratory of Brain and Cognitive Science, Institute of Biophysics, Chinese Academy of Sciences, Beijing, 100101, China; 5 Center of Medical Physics and Technology, Hefei Institutes of Physical Science, CAS, Hefei, Anhui 230031, China; 6 Centers for Biomedical Engineering, University of Science & Technology of China, Hefei, Anhui, 230027, China; 7 School of Humanities & Social Science, University of Science & Technology of China, Hefei, Anhui 230026, China

## Abstract

Novelty seeking (NS) is a personality trait reflecting excitement in response to novel stimuli. High NS is usually a predictor of risky behaviour such as drug abuse. However, the relationships between NS and risk-related cognitive processes, including individual risk preference and the brain activation associated with risk prediction, remain elusive. In this fMRI study, participants completed the Tridimensional Personality Questionnaire to measure NS and performed a probabilistic decision making task. Using a mathematical model, we estimated individual risk preference. Brain regions associated with risk prediction were determined via fMRI. The NS score showed a positive correlation with risk preference and a negative correlation with the activation elicited by risk prediction in the right posterior insula (r-PI), left anterior insula (l-AI), right striatum (r-striatum) and supplementary motor area (SMA). Within these brain regions, only the activation associated with risk prediction in the r-PI showed a correlation with NS after controlling for the effect of risk preference. Resting-state functional connectivity between the r-PI and r-striatum/l-AI was negatively correlated with NS. Our results suggest that high NS may be associated with less aversion to risk and that the r-PI plays an important role in relating risk prediction to NS.

In many circumstances, humans and other animals are naturally inquisitive and have a characteristic tendency to explore novel and unfamiliar stimuli and environments^1^. Cloninger proposed Novelty Seeking (NS) as a personality trait that refers to the tendency to be intensely exhilarated or excited in response to novel stimuli^2^.

High NS is usually a predictor of risky behaviour such as drug abuse^3^,^4^, alcohol dependence^5^ and eating disorders^6^. As such, characterising the neural correlates of NS has been a focus of cognitive neuroscience and psychiatric research. Biochemical studies have identified the noradrenergic system^7^ and the dopamine system^8^ as potential neurobiological substrates of NS. Human neuroimaging studies have reported that NS is associated with the activation elicited by emotional stimuli in the medial frontal gyrus^9^, D2 receptors in the insula^10^, inflammation^11^, visual processing^12^, and other personality traits such as harm avoidance^13^.

However, how NS influences choice preference and enhances exploration during decision making^1^ remains unclear. Regarding this question, we hypothesise that NS is related to individual risk preference and associated with the neural activation related to risk prediction during decision making, for the reasons outlined below.

First, the reward prediction and the risk prediction of a choice collectively determine the utility of the choice during decision making^14^,^15^,^16^. Risk prediction refers to the predicted reward variance (all positive)^14^,^15^,^16^,^17^, and it is weighted by (i.e., multiplied by) individual risk preference (positive or negative)^16^. Therefore, with respect to individual risk preference, a risk prediction could either enhance (with positive risk preference) or reduce (with negative risk preference) the utility of a choice. In other words, for individuals with a higher (from negative to positive) risk preference, a risk prediction would be more beneficial to the utility of a choice. Considering that risk arises from uncertainty^15^ and the uncertain nature of a novel choice^1^, a novel choice usually contains risk. Clearly, a novel choice is more attractive to individuals with high NS than to individuals with low NS. Therefore, we hypothesise that higher NS is associated with a higher risk preference. Other lines of evidence also support this idea. Previous studies have demonstrated that alcoholism is accompanied by a high risk preference^18^,^19^ and high NS^5^. A recent study reported high NS in people who participate in pathological gambling^20^. However, no direct evidence of a relationship between risk preference and NS has been provided by previous studies.

Second, risk prediction itself also impacts on the utility of a choice. Whether the neural activation associated with risk prediction is related to NS is not clear. An association between insula activation and NS^10^ has been suggested, and the insula is a risk-related brain region^21^. In addition, given our first hypothesis in the present study (i.e., NS is related to risk preference) and previous reports relating risk preference to the neural process of risk^22^,^23^, we hypothesise that the neural activation elicited by risk prediction is associated with NS during decision making.

Previous studies^1^,^24^ have reported that novel stimulus-elicited activation in the striatum^1^ and substantia nigra/ventral tegmental area (SN/VTA)^24^ is correlated with NS. However, the question of the relationships between NS and risk preference, as well as the neural processing of risk prediction, has yet to be addressed^24^. Moreover, the risk of a novel choice is confounded with novelty, and it is difficult to investigate our hypotheses. Therefore, in the present fMRI study, participants performed a probabilistic decision making task (Fig. 1). In this task, the risk is derived from the probabilistic event and not a novel choice. Therefore, the risk is pure without confounded novelty. Participants also completed the Novelty-Seeking Scale from the Tridimensional Personality Questionnaire (TPQ) to measure the personality trait of NS^25^. Using a mathematical model^15^, we quantitatively estimated individual risk preference and the predicted risk and reward of each choice^16^, as well as the difficulty of choice comparison (measured by Shannon’s entropy^26^,^27^). Then, brain regions related to the risk prediction, reward prediction and the difficulty of choice comparison were localised as regions of interest (ROIs). The relationships between the NS score, risk preference and the activation of ROIs were analysed with a Pearson correlation. Then, a partial correlation analysis was performed to verify the relationship between NS and activation associated with risk prediction after controlling for the effect of risk preference. In addition, the possible mediation effect of risk preference on the relationship between NS and the activation of risk prediction was tested with the Sobel test. Finally, because networks identified with resting state functional connectivity (rsFC) are intrinsic representations of the brain’s functional repertoire^28^,^29^, whether the rsFC in the neural network of risk prediction provides an index of individual differences in NS was also investigated.

## Results

### Behavioural Results

We simulated 1000 random data sets with a computer, and the total score for the simulated random data was 3316.58 ± 991.84 (mean ± SD, ranging from 200.00 to 6150.00). The total score for our human participants was 11265.00 ± 856.28 (mean ± SD, ranging from 9400.00 to 12375.00), and no data were discarded as a result of being outliers.

All parameters measuring the goodness of fit of the model, including the AIC, AICc, BIC and the absolute value of MLL^30^, were significantly smaller in our participant data than in random data (Table 1). The accuracy of choice prediction with the model was 90.53%±4.38% (mean and SD, ranging from 79.44% to 95.56%), which was significantly better than the 25% random level (*t* = 81.95, *p* < 0.001). These results suggest that the theoretical model is a good approximation of human decision making in this task.

The NS score was 14.57 ± 3.81 (mean ± SD, ranging from 6.00 to 22.00), and the risk preference (*l*) value was −0.15 ± 0.27 (mean±SD, ranging from −0.85 to 0.39) in our participants. The frequency distributions of the NS score and *l* are shown in Fig. S1. We found a positive correlation between risk preference and the NS score (*r = *0.555, *p = *0.001) (Fig. 2).

### Neuroimaging Results

We observed significant activation elicited by risk prediction in the right inferior parietal lobule (r-IPL), supplementary motor area (SMA), right striatum (r-striatum), left anterior insula (l-AI) and the right posterior insula (r-PI) (Fig. 3a, Table 2). Moreover, we found significant activation elicited by reward prediction in several cortical regions, including the right inferior parietal lobule, and in several subcortical brain regions, including the bilateral striatum (Fig. 3b, Table 2). We also observed significant activation elicited by the difficulty of choice comparison in the dorsal anterior cingulate cortex (dACC), anterior insula (AI), and other brain regions (Fig. 3c, Table 2).

Furthermore, we found significant correlations that exceeded the Bonferroni-corrected threshold between the NS score and activation elicited by risk prediction in four ROIs related to risk prediction, including the SMA (*r *= −0.568, *p = *0.001), r-striatum (*r = *−0.509, *p = *0.004), l-AI (*r = *−0.537, *p = *0.002) and r-PI (*r = *−0.512, *p = *0.004) (Fig. 4). For the ten ROIs related to reward prediction or the eight ROIs related to the difficulty of choice comparison, the threshold for the Bonferroni correction should have been smaller than 0.01. However, even if we set the threshold to *p* < 0.01 (the threshold for the ROIs of risk prediction), no significant correlation was found between NS and the activation elicited by reward prediction or the difficulty of choice comparison in these ROIs.

### Risk Prediction Processed in the r-PI and NS

To verify the relationships between NS and brain activation related to risk prediction, we conducted a partial correlation and mediation analysis. In the partial correlation analysis, we found that only the activation elicited by risk prediction in the r-PI showed a significant correlation with NS after controlling for the effect of risk preference (r = -0.462, p = 0.012) (Bonferroni correction with an adjusted alpha level, 0.05/4 = 0.0125). The relationships between NS and activation of the SMA (r = -0.425, p = 0.022), r-striatum (r = -0.386, p = 0.039) and l-AI (r = -0.361, p = 0.054) were not significant after controlling for risk preference.

The results of mediation analysis were consistent with those from the partial correlation, and only the activation elicited by risk prediction in the r-PI was correlated with NS without significant mediation of risk preference. The detailed results of mediation analysis are shown in the Supplementary Materials (Fig. S2 and Fig. S3).

### NS and rsFC among the ROIs

For ROIs with activation that was correlated with NS (i.e., the SMA, r-striatum, l-AI and r-PI, which were also related to risk prediction), the rsFC among them was calculated, and the group statistics of the correlation coefficient matrix are shown in Fig. S4. Then, the correlation between the rsFC among these regions and the NS score was calculated. The NS score demonstrated a negative correlation with the rsFC between the r-PI and r-striatum (*r = *-0.511, *p =* 0.004) and the rsFC between the r-PI and l-AI (*r = *-0.447, *p* = 0.009, nearly significant after Bonferroni correction) (Fig. 5). However, the NS score showed no significant correlation with the rsFC between the SMA and r-striatum (*r = *-0.433, *p *= 0.017), rsFC between the SMA and l-AI (*r = *-0.348, *p* = 0.060), rsFC between the SMA and r-PI (*r* *=* -0.081, *p* = 0.671), or the rsFC between the r-striatum and l-AI (*r* *=* -0.297, *p* = 0.111).

## Discussion

In the present study, we found that NS was positively correlated with individual risk preference. Moreover, consistent with previous studies^15^,^17^,^31^,^32^,^33^, activation elicited by risk prediction was observed in the r-IPL, SMA, r-striatum, l-AI and r-PI. The activation in the SMA, r-striatum, l-AI and r-PI showed a negative correlation with the NS score. Within these four brain regions, only the activation associated with risk prediction in the r-PI showed a significant correlation with NS after controlling for the effect of risk preference. The resting-state functional connectivity between the r-PI and r-striatum/l-AI was negatively correlated with NS.

Consistent with our first hypothesis, NS was found to be positively correlated with risk preference. Previous studies have demonstrated that alcoholism is accompanied by high risk preference^18^,^19^ and high NS^5^. These results were consistent with ours, but they did not reveal the relationship between NS and risk preference. Our results indicate a higher risk preference in people with a higher NS, suggesting that a novel choice with risk is more attractive to individuals with a higher NS.

Consistent with our second hypothesis, NS showed a negative correlation with activation elicited by risk prediction in the SMA, r-striatum, l-AI and r-PI. This suggests that NS interacts with the brain activities related to risk prediction. In addition, risk prediction, reward prediction and the difficulty of choice comparison might influence choice preference^16^ in different ways (e.g., decreased aversion to risk, changed processing of reward and different attitudes to difficulty when dealing with choice comparison). However, we did not find a significant correlation between the NS score and activation related to reward prediction or difficulty of choice comparison. This might suggest that NS predicts individual differences in risk prediction processing but not necessarily in reward prediction processing or choice comparison.

These brain activities elicited by risk prediction influence NS, and they have been reported to be important in sensation, emotion, and risk processing. For example, PI has been reported to be involved in somatosensory information processing^34^,^35^, emotional and cognitive processing^36^,^37^ and risk^15^,^38^; AI plays an important role in emotion, risk and risk prediction error^17^,^39^; the striatum responds to risk^15^, sensory information input^40^ and intuition/automatic cognitive computing^41^. This suggests that risk prediction processing may influence NS through risk sensation.

In particular, the correlation between NS and activation associated with risk prediction in these brain regions was negative. A previous study reported that for risk-seeking individuals, compared to risk-aversive individuals, risk elicited a lower activation in the striatum and insula^33^. Furthermore, we found that the correlation of NS with the rsFC between the r-PI and r-striatum/l-AI was also negative. Cox *et al.*^42^ reported that increased risk aversion was associated with a stronger rsFC between the insula and other brain regions. These results suggest that low activation of risk prediction-related brain regions and a low rsFC among these brain regions reflect decreased aversion to risk in high novelty seekers.

In brief, the present study centres around two relationships, the relationship between NS and risk preference and the relationship between NS and activation elicited by risk prediction. A positive correlation between NS and risk preference suggests that higher NS is related to higher risk preference. Alternatively, the mechanism of higher risk preference could be interpreted as preferring risk or ignoring risk. Considering the neuroimaging result showing that the correlation between NS and activation in brain regions related to risk prediction or the rsFC was negative, we speculate that high NS is related to less neural sensitivity or less aversion to risk. Therefore, both relationships collectively suggest that higher NS may be associated with less aversion to risk.

In the partial correlation analysis and supplemental mediation analysis, we found that only the activation associated with risk prediction in the r-PI showed a correlation with NS after controlling for the effect of risk preference and that only the correlation between NS and activation associated with risk prediction in the r-PI was not significantly mediated by risk preference. These results help to confirm the relationship between risk prediction processed in the r-PI and NS. Moreover, the rsFC between the r-PI and r-striatum/l-AI was negatively correlated with NS, and no significant correlation was found between NS and rsFC among other pairs of ROIs. These results consistently indicate the role of the r-PI in linking risk prediction and NS. Why is this process associated with the r-PI? The functional segregation of the insula in the anterior-posterior dimension and the possible functional significance of the rsFC between the r-PI and other ROIs are discussed below.

Numerous studies have examined the sub-regional segmentation of the insula. Most studies have reported that the AI is mostly related to self-awareness, salience detection, cognition, and other emotional/social behaviours, but the PI is mostly linked to sensory perception and motor-related functions^43^. However, a previous study demonstrated that the uncertain expectation of painful stimuli enhances responses to non-painful stimuli in the PI^44^. Another study^36^ showed that social rejection shares somatosensory representations with physical pain in the PI. Therefore, it seems that the PI is involved not only in sensory perception but also in influencing an individual’s inner state and motivation to outside stimuli. Therefore, in the present study, the PI might influence the personality trait of NS by modulating the internal state of risk sensation during risk processing. Moreover, a previous study^45^ suggested that somatosensory information is first processed in the PI and then conveyed to the AI where the emotional reaction is elaborated. Another study^46^ reported altered intrinsic functional connectivity between the AI and the PI in individuals with autism spectrum disorder, which suggests that a network involving emotional and interoceptive awareness is important for social abilities. Therefore, it seems that the PI is also involved in modulation of the affective aspect through connections with the AI. In addition, a recent study^47^ reported decreased striatal-posterior insula connectivity in cocaine-addicted individuals, and this connectivity decline predicts relapse risk and impulsivity. Considering both the previous evidence and our results, the PI might play a role in influencing the internal state of risk sensation and the motivation to outside stimuli by risk, which then manifests the personality trait of NS.

In a previous study^1^, novel stimulus-related brain activation in the striatum was found to be correlated with individual NS scores. The activation related to the reward overlapped with the striatum, and Wittmann proposed that exploration of novelty shares properties with reward processing. However, the novel stimuli in that study were complex and had more properties than the reward. Although the striatum is a reward-related brain region, it also responds to risk^15^. In particular, another study found that NS was positively correlated with reward system SN/VTA activation elicited by novel cues without expected reward^24^. In the present study, only activation elicited by risk prediction was found to be correlated with NS. Therefore, we speculate that the results of Whitman’ study could be alternatively explained as an effect of the risk prediction or novelty per se. Of course, this requires further evidence.

There are two limitations of the present study. First, the present study uses a block design, and there is no inter-trial jitter in the task implementation. Instead, we used a parametric analysis for the brain activities related to risk prediction, and the risk prediction varied trial by trial. Therefore, our task design should not greatly influence the robustness of our results. Second, although we found significant partial correlation results only in the r-PI and not in the other three brain areas, the coefficients of the other three brain regions did not show significant differences from that of the r-PI. Therefore, it is difficult to judge the functions of the other three brain regions are functional dissociated from the r-PI. In the present study, we employed two types of data analyses (the mediation analysis and the rsFC data), and both showed consistent results to those of the partial correlation, suggesting converging evidence and further supporting our conclusion. Moreover, future studies are required to reveal the different roles of these four brain areas in decision making and NS processing.

In conclusion, using the TPQ and a probabilistic decision making task, the present study examined the relationships between NS and risk-related cognitive processes. The NS score showed a positive correlation with risk preference and a negative correlation with the activation elicited by risk prediction, suggesting that higher NS might be associated with less aversion to risk. The results of partial correlation, mediation analysis and rsFC-related correlation confirm the role of the r-PI in relating risk prediction to NS. Our findings possibly offer new insight into the neural mechanisms of NS and NS-related disorders, and future studies on the PI, as well as its related neural network, might provide a new basis for the treatment of NS-related disorders.

## Materials and methods

### Participants

Thirty normal participants completed the task (3 females; mean ±SD age, 23.57 ± 2.34 years; mean ±SD years of education, 16.83 ± 2.07 years). All were graduate or undergraduate students from the University of Science and Technology of China. All participants gave informed consent in agreement with the Declaration of Helsinki. The Research Ethics Committee of the University of Science and Technology of China approved all experimental procedures. The methods were carried out in accordance with the approved guidelines.

### Task

**Figure 1:**
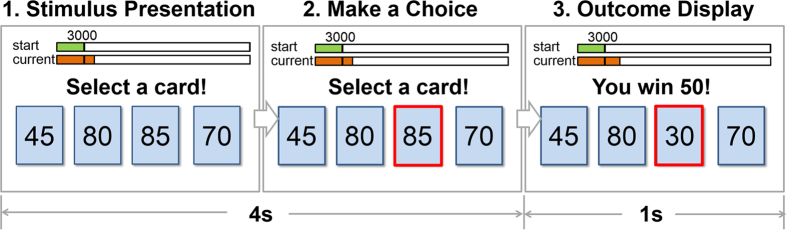
An illustration of the task. Each trial was divided into the following events: (1) the choice phase, from the moment of presentation of the four decks until the execution of the choice. Four seconds were allowed for pondering on the choice and selection. The computer would make a random choice if 4 seconds were hesitated off; (2) the outcome evaluation phase, the second card of the selected deck and the outcome were presented for 1 second on screen. After each trial, all cards were refreshed and the next trial began immediately without inter-trial intervals (ITIs).

**Figure 2:**
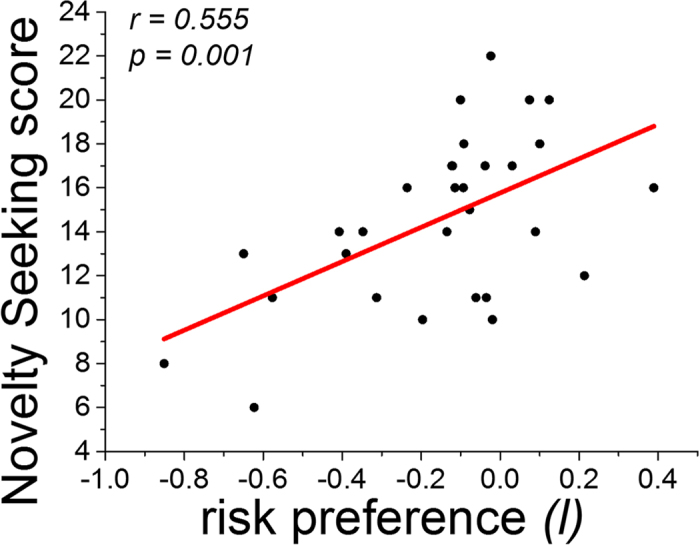
Behavioural result. The NS score was positively correlated with risk preference.

**Figure 3:**
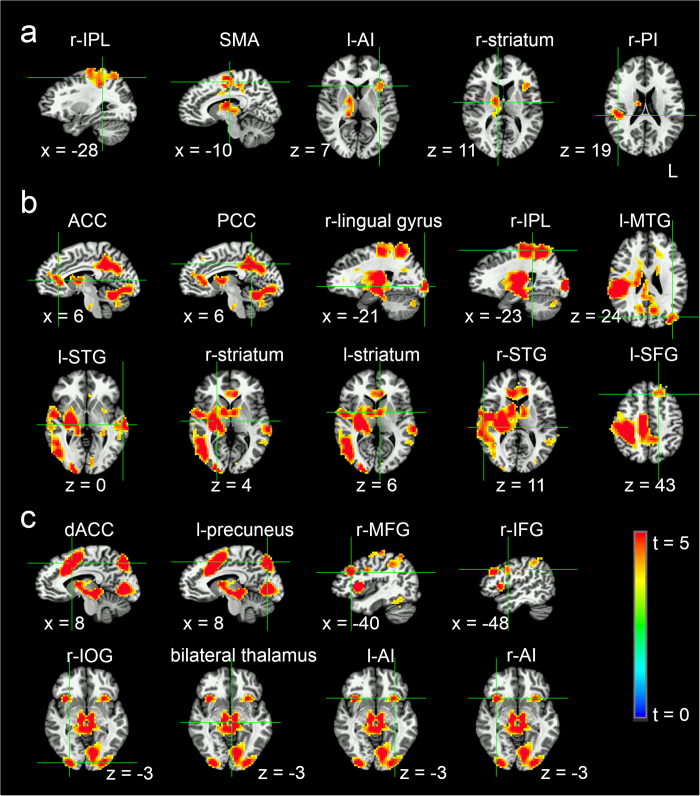
Localised ROIs. Brain regions responding to *a,* the risk prediction; *b,* the reward prediction; *c,* the difficulty of choice comparison.

**Figure 4:**
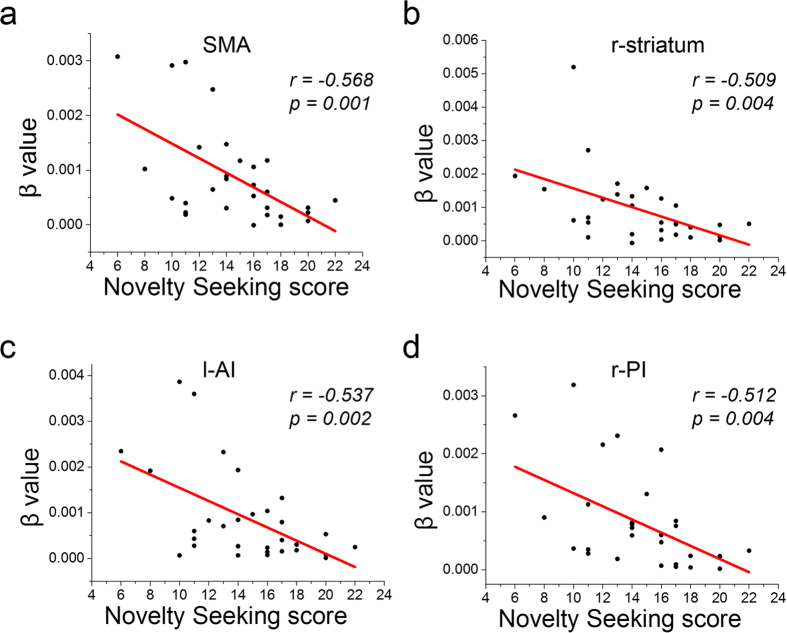
Negative correlations between NS and activation elicited by risk prediction in *a,* the SMA; *b,* the r-striatum; *c,* the l-AI; *d,* the r-PI.

**Figure 5:**
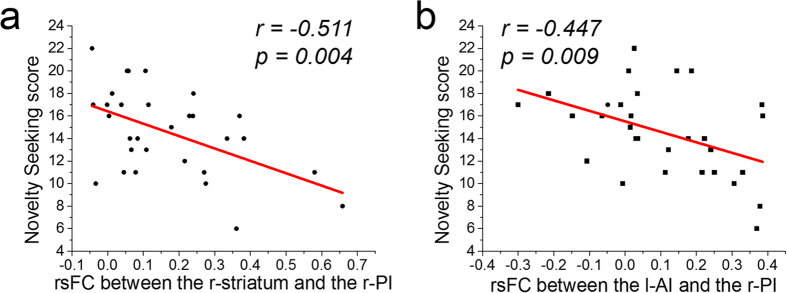
The correlation between rsFC and NS. The rsFC between r-PI and l-AI/r-striatum was negatively correlated with the NS score.

The task was modified from a former study^15^. There were 180 trials in total. In each trial, four decks (Deck A, B, C, and D) were presented horizontally on the computer screen. A number randomly selected from an arithmetic sequence (from 0 to 100, step 5) was explicitly presented on each card. The participants chose one deck among the four decks. If the number on the first card of the selected deck was larger than that on the second card of the deck, the participant would win a certain number of points. Otherwise, the participant would lose a certain number of points (Fig. 1). Specifically, the participants would win 100 points or lose 125 points by selecting Deck A or B and would win 50 points or lose 25 points by selecting Deck C or D. The numbers were pseudo-randomised and were the same across all participants. In each deck, the numbers on adjacent cards were never the same. In each trial, the number on Deck A was different from that on Deck B because of their shared possible winning and losing, the number on Deck C was different from that on Deck D. The number on Deck A could be the same as that on Deck C or D, etc.

### Procedure

Participants were clearly informed the instructions regarding the task described above and were informed that the experiment did not involve deception. Participants were also briefed on the payment, including the fact that the payment would be sensitive to the task performance. Subsequently, participants completed the training session for the task outside the scanner, which lasted ~5 min and did not involve any payment. During the training session, each participant performed a short version of the task to become familiar with it. Then, after a ~10 min break and before entering the MR scanner, all participants were told to keep their heads steady during all scans. One 8 min functional scan for resting-state fMRI data was performed while participants kept their eyes closed, followed by three functional scans consisting of 180 trials for fMRI data collection during the task; each scan lasted for 7 min. There was an interval of ~1 min between each scan. Each scan consisted of 3 task blocks separated by a 30 s-resting (fixation) block, and each task block contained 20 trials. Participants received the accumulated outcomes from the task in the scanner plus the base endowment of 30 RMB (approximately 4.8 U.S. dollars). Finally, the amount of the payment participants received was between 94.00 RMB (approximately 15.06 U.S. dollars) to 123.75 RMB (approximately 19.83 U.S. dollars).

### Novelty-Seeking Scale

All participants completed the Tridimensional Personality Questionnaire, which contains three subscales including the Novelty Seeking Scale. In this study, we only analysed data from the Novelty-Seeking Scale, which contains 34 true-false questions, and higher scores reflect greater NS. Cronbach’s Alpha value of internal consistency measurement was 0.698. The Novelty-Seeking scale has been widely used in the Chinese population; it has good psychometric properties in the literature and is reported to be cross-culturally robust^25^,^48^.

### Data Acquisition

All MRI scans were collected on a Siemens Magnetom Trio 3.0-T scanner (Siemens Medical Solutions, Erlangen, Germany) in the Anhui Provincial Hospital. A circularly polarised head coil was used, with foam padding to restrict head motion. Functional images, including the task-related fMRI data and the resting state fMRI data, were acquired with a T2*-weighted echo-planar imaging sequence (TE = 30 ms, TR = 2000 ms, FOV = 240 mm, Matrix = 64 × 64, flip angle = 85°) with 33 axial slices (no gaps, voxel size: 3.75 × 3.75 × 3.7 mm3) covering the whole brain. Corresponding high-resolution T1-weighted spin-echo (for anatomical overlay) images and three-dimensional gradient-echo (for stereotaxic transformation) images were also collected (TR = 1900 ms; TE = 2.26 ms; TI = 900 ms; 1 mm isotropic voxel; 250 mm field of view (FOV)).

### Behavioural Data Modelling

The utility of each choice was measured in combination with its predicted reward and risk (variance of the reward^15^,^17^). The algorithms were implemented with MATLAB (version 7.6.0.324, The Mathworks Inc, Natick, MA).

In accordance with previous studies of risk during decision making^15^,^17^, in this study, 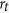 denoted the reward and 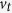 denoted the expected reward based on the number on the first card, i.e.,


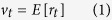


The prediction error equals 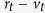. 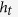 denotes the risk prediction, the risk prediction is the expected size-squared of this prediction error, namely, the variance,


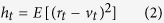


Based on the reward prediction and the risk prediction, the utility of choice i at trial t could be estimated^16^.


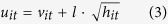


where *l* is the risk preference^49^.


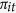
 is the probability to select the choice i in trial t; 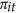 was calculated with the softmax rule^16^ (n=4):


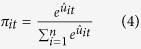


The algorithm was fitted to the data from the 180 decisions (trials) made by each participant. The risk preference *(l)* was estimated for each participant by maximising the log transformed likelihood^16^:


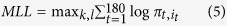


For it, the deck chosen at trial t, t = 1…, 180, i_t_ ∈{1, 2, 3, 4}, and 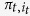, is the probability of selecting deck i_t_ in trial t. The global maximum was a search with 1000 steps and for 4 restarts; the parameter constraint was *l* ∈[−1,1].

Shannon’s Entropy^26^,^27^ was computed to measure the difficulty of choice comparison in a trial:


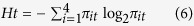


In summary, through behavioural modelling, the reward prediction, risk prediction and difficulty of choice comparison were estimated. At the same time, the risk preference (*l*) was also estimated.

### Behavioural Data Analysis

We simulated 1000 random data sets of the task with a computer. We modelled the random data with the same algorithm as that used for the participants’ data. The goodness of fit of the model was evaluated using the maximum likelihood (MLL)^30^, Akaike information criterion (AIC) values, AICc (the corrected form of the AIC), and Bayesian exceedance probability (BIC)^30^. The statistics of the model for participant data were compared with those for the random data with an independent-samples t-test. In addition, the probabilities of choosing the four decks in a trial were obtained in the modelling. The deck with maximal probability was considered the predicted choice by our model. The prediction accuracy of the model was compared with the random level (25%) with an independent-samples t-test. Finally, the Pearson correlation between individual risk preference and the NS score was also computed.

### fMRI Data Analysis

#### Preprocessing for task-related fMRI data

The imaging data were processed with AFNI^50^. Linear and quadratic detrending was first applied to each participant’s raw data, then it was corrected for temporal shifts between slices and head motion, spatially smoothed with a Gaussian kernel (full width at half maximum = 8 mm), and temporally normalised. Head movement in the MRI scanner for all data was less than 2 mm or 2 degrees in displacement or rotation.

#### Localising of regions of interest (ROIs)

After preprocessing of the task-related fMRI data, a General Linear Model (GLM) was used to localise the regions of interest (ROIs) related to the risk prediction, reward prediction, and difficulty of choice comparison for each participant. This analysis included the regressors constructed from trial-by-trial estimates of the risk prediction, reward prediction, and difficulty of choice comparison. These regressors were convolved by the gamma function to approximate the haemodynamic response of the brain to a stimulus. The regression analysis also included six regressors for head motion.

The resultant activation (z-transformed β-value) of each regressor in each participant was transformed to the Talairach space and then entered into a group-level one-sample t test. Responses were identified if they survived whole brain correction for family-wise error at a cluster-level threshold of p < 0.01 (e.g., risk prediction, cluster size > 2430 mm^3^) and a voxel-level threshold of p < 0.001. Clusters in which the activation showed a positive correlation with risk prediction were defined as ROIs related to risk prediction. ROIs related to the reward prediction or difficulty of choice comparison were obtained in a similar manner.

#### Correlation between NS and activation of ROIs

For each risk prediction-related ROI, the averaged activation of voxels elicited by risk prediction (z-transformed β-value) within this ROI was calculated for each participant. The correlation coefficient between the activation and his/her NS score was calculated with a Pearson correlation. Because there were five ROIs related to risk prediction in total, the significance level for each correlation coefficient was performed with multiple comparisons correction (Bonferroni correction with an adjusted alpha level, 0.05/5 = 0.01). The same procedure was applied to ROIs related to reward prediction and the difficulty of choice comparison.

#### Partial correlation and mediation effect of the risk preference

One of the main findings in this study was the correlation between neural activation associated with risk prediction and NS. However, consistent with previous reports^22^,^23^, our fMRI results showed a correlation between the neural activation elicited by risk prediction and risk preference. Our behavioural results showed that risk preference was in turn associated with the NS score. Therefore, it is reasonable for us to suspect that the neural basis of risk prediction influences NS via a mediation effect of individual risk preference. Because it is rational to consider neural activation the biological basis relating an individual trait (e.g., risk preference) to another personality trait (e.g., Novelty Seeking), we used a directed mediation analysis (See details in Supplementary Materials). To verify the relationship between the activation related to risk prediction and NS, an analysis of partial correlation between NS and the brain activation associated with risk prediction after controlling for individual risk preference was also performed with multiple comparisons correction (Bonferroni correction with an adjusted alpha level, 0.05/4 = 0.0125).

#### Correlation between rsFC and NS

Resting-state fMRI data processing was also conducted with AFNI. Initial processing included linear and quadratic detrending, correction for temporal shifts between slices and head motion, spatially smoothing with a Gaussian kernel (full width at half maximum = 8 mm), and temporal normalisation. We further performed a regression of all voxels’ time serials with regard to 6 motion parameters, and data scrubbing was performed following the method of Power and colleagues^51^, excluding any volume with a framewise-dependent value exceeding 0.5. During the scrubbing step, the number of removed volumes ranged from 0 to 21 (less than 10% of all the 240 volumes) among all participants. Then, bandpass temporal filtering (0.01–0.08 Hz) was performed on the residual signals to obtain the low-frequency fluctuation. Next, to further reduce nuisance signals, we regressed out the average signals in the white matter and the CSF. The mask of white matter for each participant was determined from the high-resolution structural image using the FAST segmentation program of Functional MRI of the Brain software library (www.fmrib.ox.ac.uk). The CSF mask for each participant was manually drawn according to the anatomical boundaries of the cortical structures of a standardised Talairach atlas brain, transformed onto the image space of the individual, and then modified according to the cortical structures of the individual brain by referencing to the anatomical boundaries in the high resolution three-dimensional structural image. These nuisance signals were used to account for fluctuations that were likely not relevant to neuronal activity. The resultant resting-state fMRI data were then subjected to functional connectivity analysis^52^.

ROIs that were significantly correlated with the NS score were selected as seed regions. Then, for each participant, the seed region defined in Talairach space was transformed to his/her original image space. The preprocessed resting-state fMRI time series were averaged within each seed region. Correlation coefficients (r values) were calculated among averaged time series of the seed regions and then transformed to Fisher z values. Then, the correlation between the z values and the NS scores was calculated. We performed a multiple comparisons correction with a threshold of 0.008 (Bonferroni correction with an adjusted alpha level, 0.05/6 = 0.008).

## Additional Information

**How to cite this article**: Wang, Y. *et al.* Novelty seeking is related to individual risk preference and brain activation associated with risk prediction during decision making. *Sci. Rep.* **5**, 10534; doi: 10.1038/srep10534 (2015).

## Supplementary Material

Supplementary Information

## Figures and Tables

**Table 1:** Goodness of fit of the model for our participants’ data and computer-simulated random data.

	**participants’ data**	**random data**	**independent t-test**	**p value**
AIC	2.62±0.30	4.78±0.02	t = −212.49	p < 0.001
AICc	2.77±0.30	4.79±0.02	t = −198.83	p < 0.001
BIC	4.03±0.30	9.69±0.02	t = −557.475	p < 0.001
MLL	0.31±0.15	1.39±0.01	t = −212.49	p < 0.001

MLL, maximum likelihood; AIC, Akaike information criterion values; AICc, the corrected form of AIC; and BIC, Bayesian exceedance probability

**Table 2:** Brain regions responding to the risk prediction, the reward prediction, and the difficulty of choice comparison, all survived whole-brain correction for familywise error at a cluster-level threshold of p < 0.01 and a voxel-level threshold of p < 0.001.

**ROI**	**Brain region**	**Voxels**	**x**^a^	**y**	**z**
risk prediction					
r-IPL	right inferior parietal lobe	726.0	−40.5	31.5	35.5
SMA	supplementary motor area	310.0	−13.5	−4.5	32.5
r-striatum	right striatum	193.0	−12.7	13.2	10.8
l-AI	left anterior insula	112.0	34.2	−18.1	6.8
r-PI	right posterior insula	92.0	−44.2	28.1	23.1
reward prediction					
r-striatum	right striatum	1133.0	−10.5	−7.5	−6.5
l-striatum	left striatum	195.0	10.0	1.2	12.0
PCC/precuneus	posterior cingulate cortex/precuneus	1791.0	−5.0	36.5	39.7
r-IPL	right inferior posterior lobe	1440.0	−36.6	31.7	48.7
r-STG	right superior temporal gyrus	2600.0	−44.7	28.5	2.6
l-STG	left superior temporal gyrus	2369.0	32.6	36.5	−13.8
ACC	anterior cingulate cortex	412.0	3.3	−29.3	14.9
l-SFG	left superior frontal gyrus	139.0	21.1	−30.7	43.2
r-lingual gyrus	right lingual gyrus	134.0	−21.2	91.2	0.2
l-MTG	left middle temporal gyrus	132.0	35.4	77.5	25.0
choice difficulty					
dACC	dorsal anterior cingulate cortex	2475.0	8.7	−6.0	43.6
l-precuneus	left precuneus	2294.0	13.8	67.4	23.2
thalamus	bilateral thalamus	834.0	−1.5	18.7	0.2
l-AI	left anterior insula	306.0	31.8	−14.9	7.2
r-AI	right anterior insula	279.0	−35.4	−14.0	5.4
r-MFG	right middle frontal gyrus	258.0	−36.8	−29.4	30.9
r-IOG	right inferior occipital gyrus	114.0	−27.7	87.1	−3.9
r-IFG	right inferior frontal gyrus	89.0	−47.0	−0.8	31.9

^a^Coordinates in Talairach space (x, positive left and negative right; y, positive posterior to negative anterior; z, positive superior and negative inferior).
