# Supplementary material for: Novelty seeking is related to individual risk preference and brain activation associated with risk prediction during decision making
Source: Sci Rep. 2015 Jun 11;5:10534. doi: 10.1038/srep10534 (PMC4464254; doi:10.1038/srep10534)
Supplement: Supplementary Information [file srep10534-s1.doc]

**Novelty seeking is related to individual risk preference and**

**brain activation associated with risk prediction during decision making**

Ying Wang, Lizhuang Yang, Feng Gu, Xiaoming Li, Rujing Zha, Zhengde Wei, Yakun Pei,

Ying Liu, Yifeng Zhou, Xiaochu Zhang

**Inventory of Supplemental Information**

**Supplemental Figures**

- Figure S1: Frequency distributions of the Novelty Seeking score and individual risk preference
- Figure S2: Results of the mediation analysis, and the solid lines highlight the tested pathway
- Figure S3: The correlation between the activation elicited by risk prediction and risk preference within an independent group of participants
- Figure S4: Resting-state functional connectivity matrix

**Supplemental Materials for Mediation Analysis**


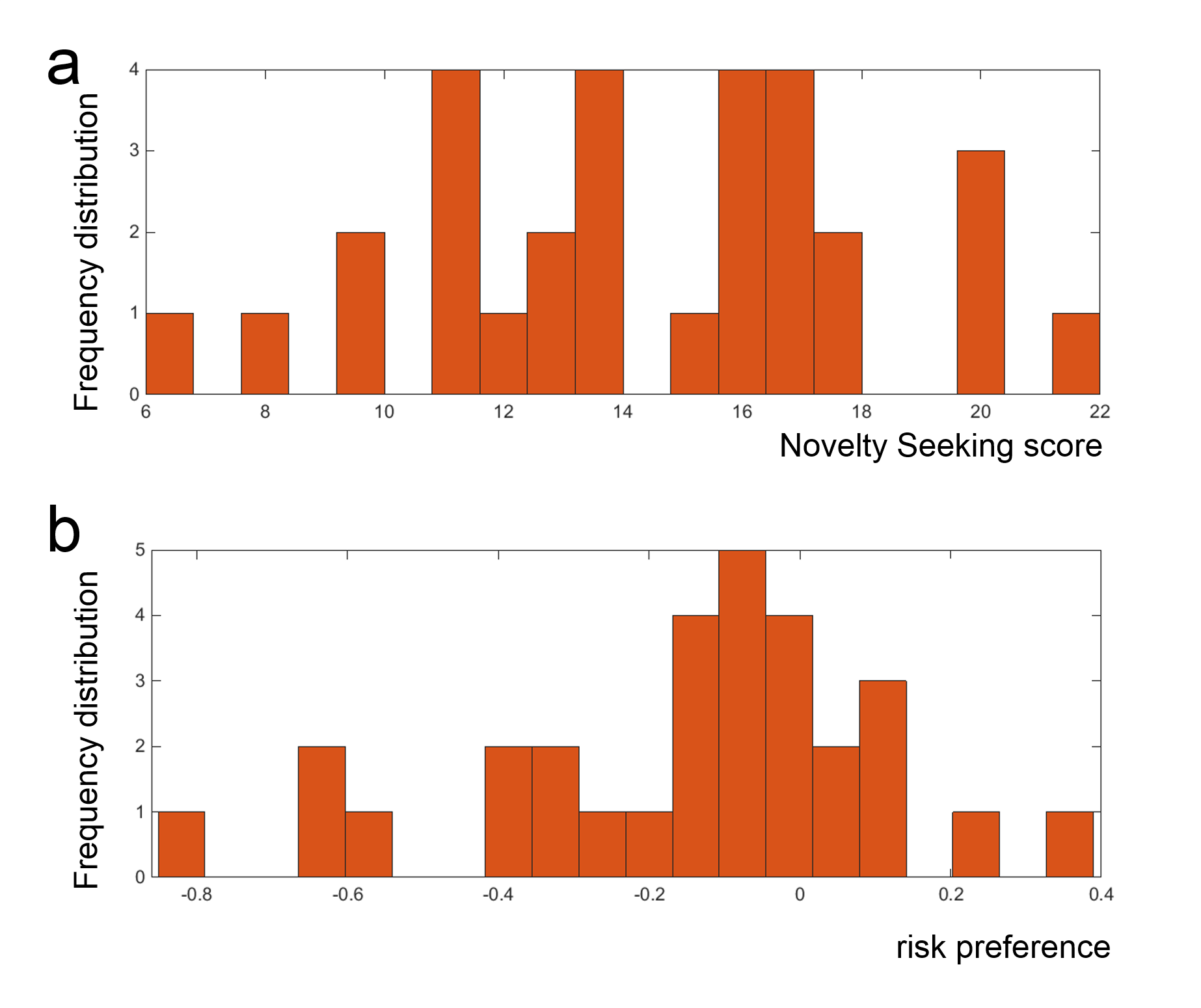


**Supplementary Figure 1. Frequency distributions of the Novelty Seeking score and risk preference.** *a,* the Novelty Seeking score; *b,* risk preference.

**
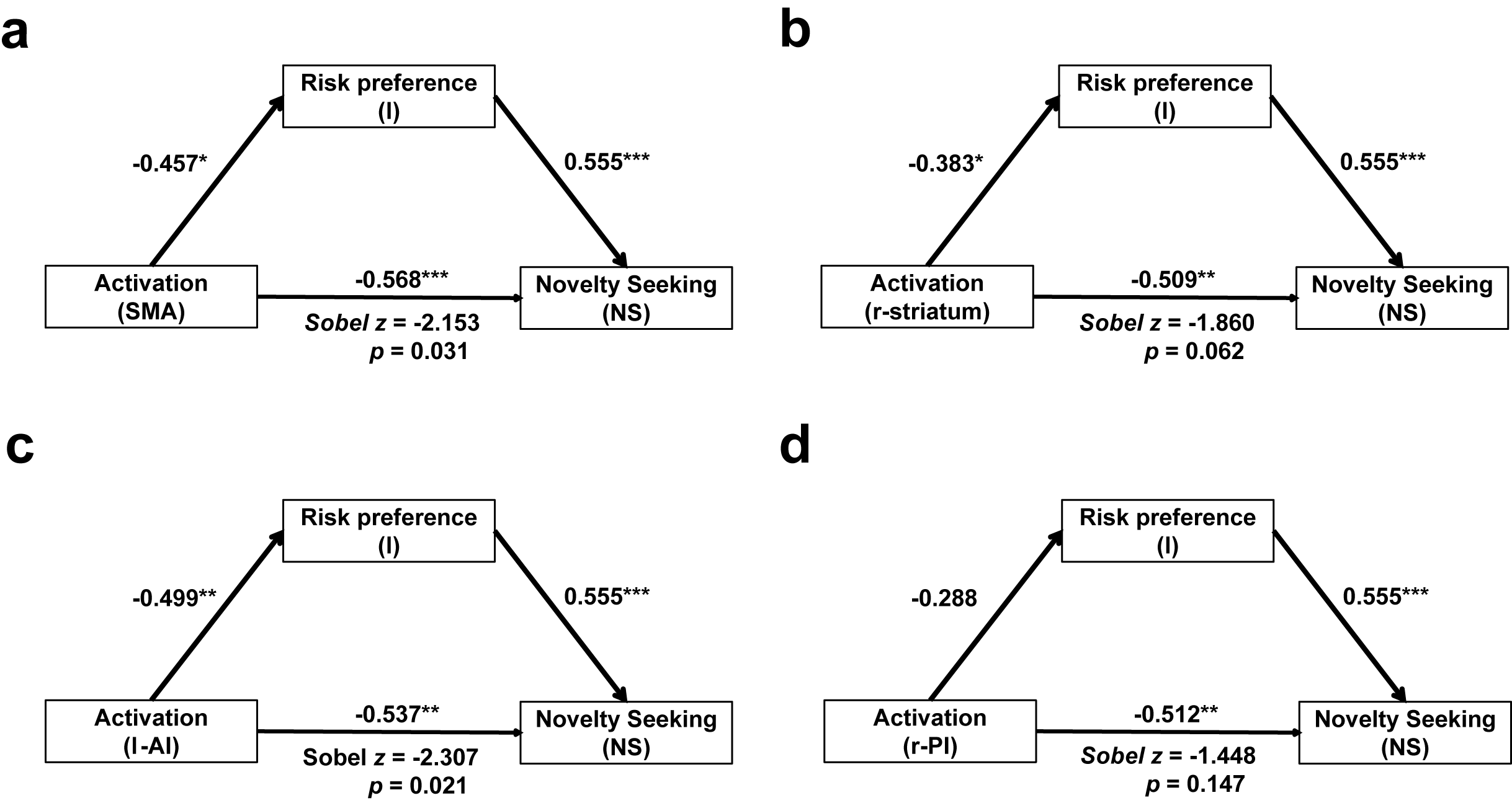
Supplementary Figure 2.** **Results of the mediation analysis, and the solid lines highlight the tested pathway.** *a-c,* these figures showed significant correlations between the risk preference and the activation elicited by risk prediction in the SMA (*r = -0.457*, *p* = 0.011), r-striatum (*r = -0.383*, *p* = 0.037), and the l-AI (*r =- 0.499*, *p* = 0.005), and demonstrated a mediation effect of risk preference on the correlation between NS and the activation in the SMA, the r-straitum, and the l-AI; *d,* the figure showed a non-significant correlation between the risk preference and the activation elicited by risk prediction in the r-PI (*r = -0.288*, *p* = 0.123), and demonstrated no significant mediation effect of risk preference on the correlation between NS and the activation in the r-PI. Asterisks indicate significant associations (*, *p* < 0.05, **, *p* < 0.01, ***, *p* < 0.001).


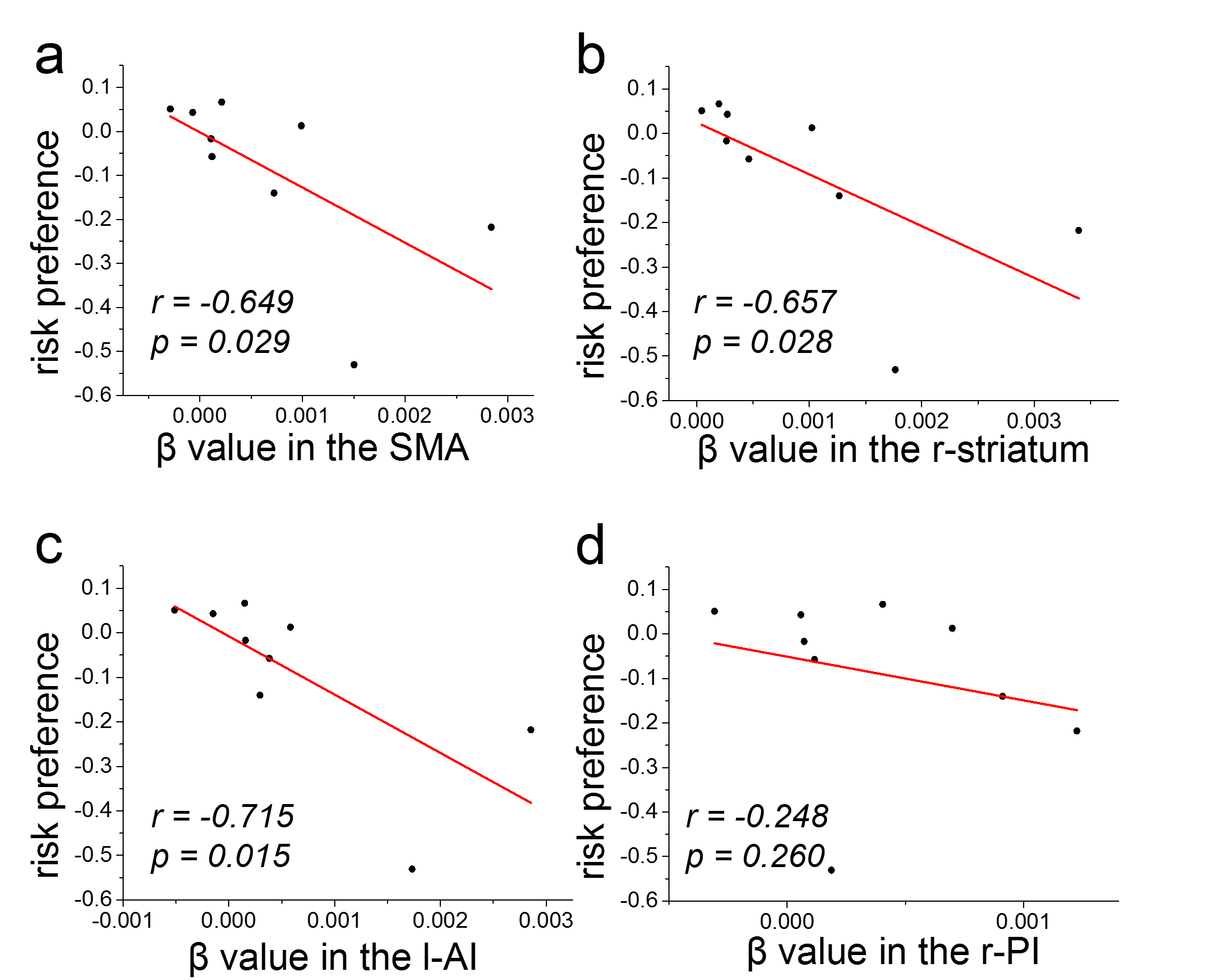


**Supplementary Figure 3. The correlation between the activation elicited by risk prediction and risk preference within an independent group of participants.** *a-c,* there was a significant correlation between the risk preference and the activation related to the risk prediction in the SMA, r-striatum, and the l-AI; *d,* there was no significant correlation between the risk preference and the activation elicited by risk prediction in the r-PI.


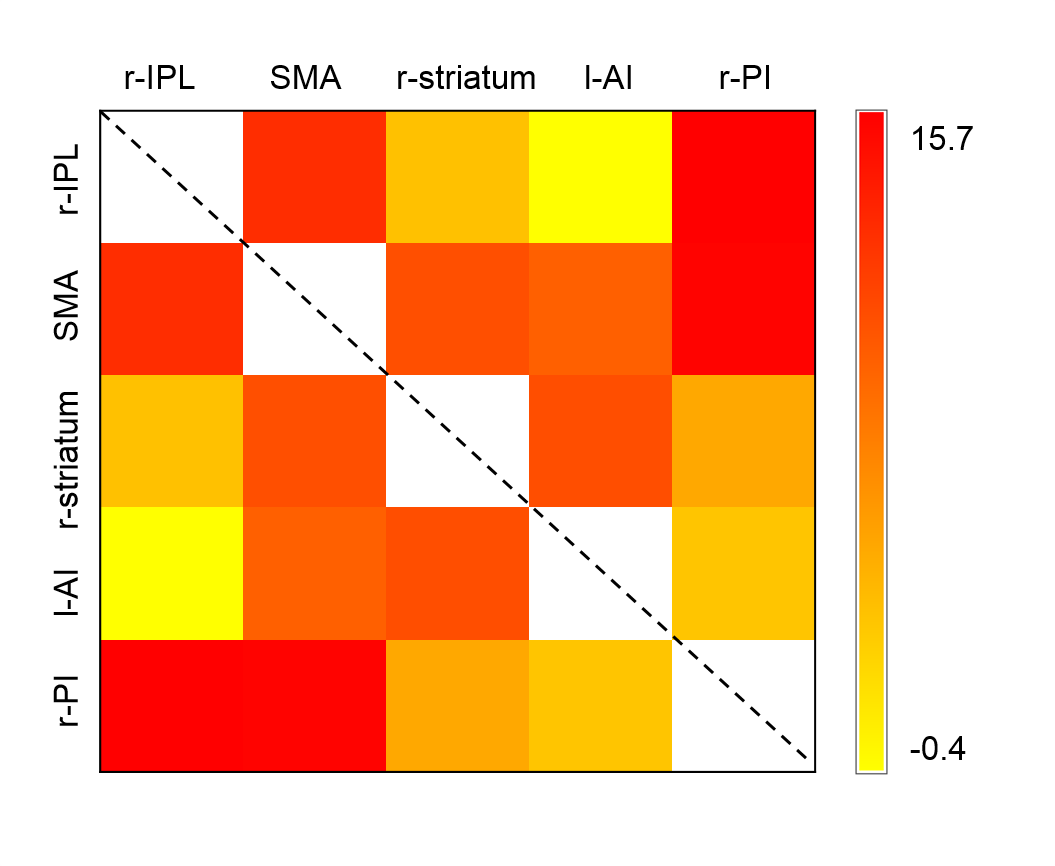


**Supplementary Figure 4. Resting-state functional connectivity matrix.** Color bar demonstrated the range of the group statistics (t value) of the correlation coefficient.

**Supplemental Materials for Mediation Analysis**

Although the activation associated with risk prediction in the SMA, r-striatum, and the l-AI was negatively correlated with the NS score, the activation was also correlated with individual risk preference, which in turn was associated with the NS score (Fig. S2a-c). Together, these results indicate a possible indirect effect of the activation related to risk prediction in these ROIs on NS.

Standardised regression coefficients for the associations among all variables (activation associated with risk prediction in risk prediction-related ROIs, risk preference and NS) were calculated with Matlab, and the Sobel test was carried out with the free Sobel Test Calculator on line (http://www.danielsoper.com/statcalc3/calc.aspx?id=31). For the mediation analysis, we didn’t do multiple comparisons correction, because we want to isolate the correlation between activation and NS that is not mediated by risk preference.

The Sobel (mediation) test demonstrated an indirect or marginally indirect effect of activation in the SMA (*z* = -2.153, *p =* 0.031), r-striatum (*z =* -1.860, *p =* 0.062), and the l-AI (*z* = -2.307, *p =* 0.021) on NS (Fig. S2a-c). The activation in the r-PI was not significantly correlated with individual risk preference (Fig. S2d), and the mediation analysis demonstrated that only the r-PI showed no significant evidence for this path *(z* = -1.448, *p =* 0.147) (Fig. S2d).

However, in the 30 participants data, the risk preference variable was derived from the same data that generated risk prediction-related ROIs, there was potential lack of independence between the activation in these ROIs and the risk preference. Therefore, an independent group of ten normal participants (2 females; mean ± SD age, 23.50 ± 1.57 years; mean ± SD years of education, 17.00 ± 1.26 years) were recruited to test the relationship between the risk preference and the activation associated with risk prediction in the risk prediction-related ROIs. Behavioral data of these participants were modelled. For fMRI data analysis, we took a ROI-based approach. ROIs included the four brain regions (the SMA, r-striatum, l-AI and the r-PI) defined with the 30 participants’ data. Among the independent group of participants, one data was excluded because of extremely large risk preference (three times SD larger than the mean). Because of the negative correlation in the 30 participants, we hypothesise that, in the independent group of participants, there should be a negative correlation between the activation associated with risk prediction in these four ROIs and individual risk preference. Therefore we took a one-tailed correlation analysis, and found that the activation associated with the risk prediction in the SMA (*r* = -0.649, *p* = 0.029), r-striatum (*r* = -0.657, *p* = 0.028) and l-AI (*r* = -0.715, *p* = 0.015) exhibited a negative correlation with individual risk preference (Fig. S3a-c). Consistent with the results of the mediation analysis with 30 participants, no significant correlation between activation related to risk prediction in r-PI and risk preference was found (*r* = -0.248, *p* = 0.260) (Fig. S3d).
